# Characteristics of fish-bone foreign bodies in the upper aero-digestive tract: The importance of identifying the species of fish

**DOI:** 10.1371/journal.pone.0255947

**Published:** 2021-08-17

**Authors:** Tadahisa Shishido, Jun Suzuki, Ryoukichi Ikeda, Yuta Kobayashi, Yukio Katori

**Affiliations:** Department of Otolaryngology, Head and Neck Surgery, Tohoku University Graduate School of Medicine, Sendai, Miyagi, Japan; Yale-New Haven Hospital, UNITED STATES

## Abstract

**Background:**

Fish bones are common foreign bodies in the upper aero-digestive tract, but their clinical features in relation to fish species have not been confirmed. We aimed to clarify the clinical characteristics of fish-bone foreign bodies and their location and removal methods depending on the fish species.

**Study design:**

Retrospective, observational, monocentric study.

**Methods:**

From October 2015 to May 2020, 368 patients visited the Department of Otolaryngology-Head and Neck Surgery at Tohoku University Hospital complaining of dysphagia, sore throat, or pharyngeal discomfort after eating fish. We analyzed the patients’ sex and age distribution, foreign-body location, type of the fish, and the techniques used for removing the foreign body.

**Results:**

Fish bones were confirmed in the upper aero-digestive tract in 270 cases (73.4%), of which 236 (87.4%) involved fish-bone foreign bodies in the mesopharynx. The most frequently involved site was the palatine tonsil (n = 170). Eel was the most frequently observed fish species (n = 39), followed by mackerel (n = 33), salmon (n = 33), horse mackerel (n = 30), and flounder (n = 30). Among the 240 cases in which the bones did not spontaneously dislocate, 109 (45.4%) were treated by endoscopic removal (103 cases) or surgery (6 cases). In pediatric cases (<12 years old), almost all fish bones were found in the mesopharynx (138/139, 99.3%), and 31 cases (22.3%) required endoscopic removal. Flounder fish bones were often lodged in the hypopharynx and esophagus (9/30, 30%), hindering spontaneous dislocation and frequently necessitating endoscopic or surgical removal (19/29, 65.5%).

**Conclusion:**

The characteristics of fish-bone foreign bodies differed depending on the fish species. Flounder bones were often stuck in the hypopharynx and esophagus and were likely to require more invasive removal methods. Confirming the species of the fish could facilitate appropriate diagnosis and treatment of fish-bone foreign bodies.

## 1. Introduction

Fish bones in the upper aero-digestive tract are a common presentation in the emergency department and in otolaryngology clinics, especially in countries with high rates of fish consumption, such as Asian, Mediterranean, and other coastal countries [1–4]. Whereas food bolus impaction (in adults) and coins (in children) are the major foreign bodies in the upper aero-digestive tract in Western countries [5–7], fish bones have been reported to account for the majority of upper aero-digestive tract foreign bodies (about 50%-90% of total foreign bodies) in Asian countries [1–3, 8–10]. The age distribution of fish-bone foreign bodies varies, and children, especially those aged 2–4 years, and middle-aged adults show the highest prevalence [1, 2, 11]. Among adults, fish-bone foreign bodies are more likely to occur in people in their 40s or older because of deterioration of swallowing function [11, 12]. The common impaction sites of fish bones include the palatine tonsils, lingual tonsils, vallecula of the epiglottis, and pyriform sinus. The most frequent impaction sites in children are the palatine tonsils because of the bulkier swelling of the tonsils and anatomical narrowing of the pharynx in comparison with adults [2, 3]. Although fish-bone foreign bodies are not frequently associated with severe complications, they can cause several life-threatening complications such as perforation of the esophagus, deep neck infection/abscess, mediastinitis/mediastinal abscess, and aortoesophageal fistula [1, 2, 13]. Therefore, appropriate diagnosis and prompt treatment are required to manage fish-bone foreign bodies in the upper aero-digestive tract. The existing removal methods include direct removal from the oral cavity, removal using transnasal flexible laryngoscopy under local anesthesia, and surgery with rigid esophagoscopy or direct laryngoscopy under general anesthesia [1, 2, 14].

Information regarding the differences in the predilection sites of bone impaction and the need for invasive procedures depending on the types of fish would facilitate appropriate preparation of treatment procedures. Moreover, since prevention remains the most effective approach to manage foreign-body cases [3], public understanding of the risk of fish-bone foreign bodies for different fish species is essential. However, few reports have assessed the differences in the clinical characteristics of fish-bone foreign bodies in relation to the fish species. To address this issue, we retrospectively investigated the clinical characteristics of fish-bone foreign body patients and analyzed the differences in the predilection sites and removal methods among various fish species.

## 2. Materials and methods

### 2.1 Patients

**Fig 1:**
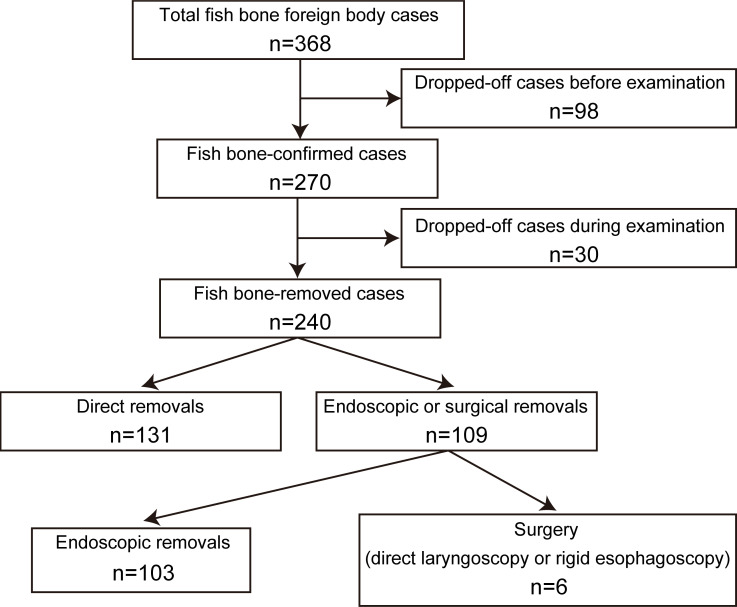
Patient enrolment and the treatment of fish-bone foreign bodies.

According to the International Statistical Classification of Diseases and Related Health Problems, Tenth Revision (ICD-10), we selected patients with the following diagnoses: “Foreign body in pharynx,” “Foreign body in respiratory tract,” or “Foreign body in esophagus.” We screened all patients who directly visited or were referred to the Department of Otolaryngology-Head and Neck Surgery at Tohoku University Hospital with any of these diagnoses from the medical records system of our hospital from October 2015 to May 2020. We selected 368 patients who complained of dysphagia, sore throat, or pharyngeal discomfort after eating fish food that was known or suspected to contain fish-bone foreign bodies. We conducted a retrospective chart review of the patients and recorded the patients’ sex and age distribution, location of the fish-bone foreign body, type of fish, and the technique used for removal of the fish bone. We excluded 98 cases in which the foreign bodies could not be identified by doctors and the symptoms had resolved at the time of visit, and the remaining 270 cases were included in the main analyses (Fig 1). Of the 270 cases in which fish bones were initially visually confirmed at a consulting room by doctors, the impacted fish bones were dislocated during further history-taking and examination, including oral inspection and flexible videoendoscopy, in 30 cases.

### 2.2 Examination and treatment

Examination and treatment were performed basically as follows. (1) When a fish bone was identified in the oral cavity or the mesopharynx, it was removed by “direct removal” using a tongue depressor and several types of forceps without the use of endoscopes. (2) In other cases, a flexible videoendoscopy(OTV-S190 and ENF-V2, Olympus, Tokyo, Japan) was performed to observe the mesopharynx and hypopharynx. When a fish bone was located, it was removed by “endoscopic removal” using a flexible videoendoscope with an instrument channel (OTV-S190 and ENF-VT2, Olympus, Tokyo, Japan) allowing passage of a grasping forceps under local anesthesia [15, 16]. (3) When the flexible endoscopic examination failed to detect the foreign body and residue in the hypopharynx or the esophagus was still suspected, we performed a non-contrast computed tomography (CT) scan (GE BrightSpeed Elite 16, GE Healthcare, Chicago, Illinois, USA). (4) If a foreign body was detected by CT scan, it was removed via “endoscopic removal” by gastroenterologists. (5) In patients with a high risk of esophageal perforation or in cases of unsuccessful endoscopic removal, otolaryngologists performed the removal using a direct pharyngoesophagoscope under general anesthesia or via external cervical incision, and we refer to these removal techniques as “surgery”.

### 2.3 Classification of fish species

Given the wide variety of fish species, we categorized some of them according to the order, family, and size of the fish by referring to the Web Fish and Shellfish Directory (https://www.zukan-bouz.com/) as follows: Eel (Japanese eel, conger eel, and daggertooth pike conger), Flounder (right-eye flounder and left-eye flounder), and Amberjack (Japanese amberjack and great amberjack). The fish that did not comprise 10 cases were categorized by size as Others (large) if they generally exceeded 30–40 cm or as Others (small).

### 2.4 Ethics

This study was approved by the Institutional Review Board at Tohoku University Graduate School of Medicine (2020-1-1023). All data were analyzed anonymously, and the IRB waived the requirement for informed consent.

### 2.5 Statistical analysis

Statistical analyses were conducted using JMP Pro 15 (SAS Institute Inc.). All data are presented as means ± standard deviation (SD). The Wilcoxon rank-sum test and Fisher exact test were used to compare the data in different groups; *P* < 0.05 was considered statistically significant.

## 3. Results

**Fig 2:**
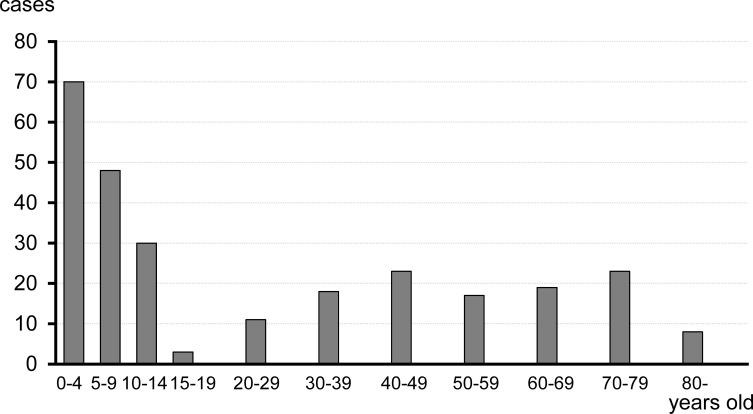
Patients’ age distribution. Patients aged 0–4 years formed the largest group and represented 25.9% of the study population.

The 270 patients with confirmed fish-bone foreign bodies included 124 males and 146 females and were aged 1–93 years (mean age, 27.2 ± 27.1 years; median age, 11 years; interquartile range [IQR], 4.0 to 49.5 years). In Fig 2, the patients’ ages show a biphasic distribution with a high peak in children and a gradual peak in mature to old age. Patients aged 0–4 years formed the largest age group and represented 25.9% of the study population.

**Fig 3:**
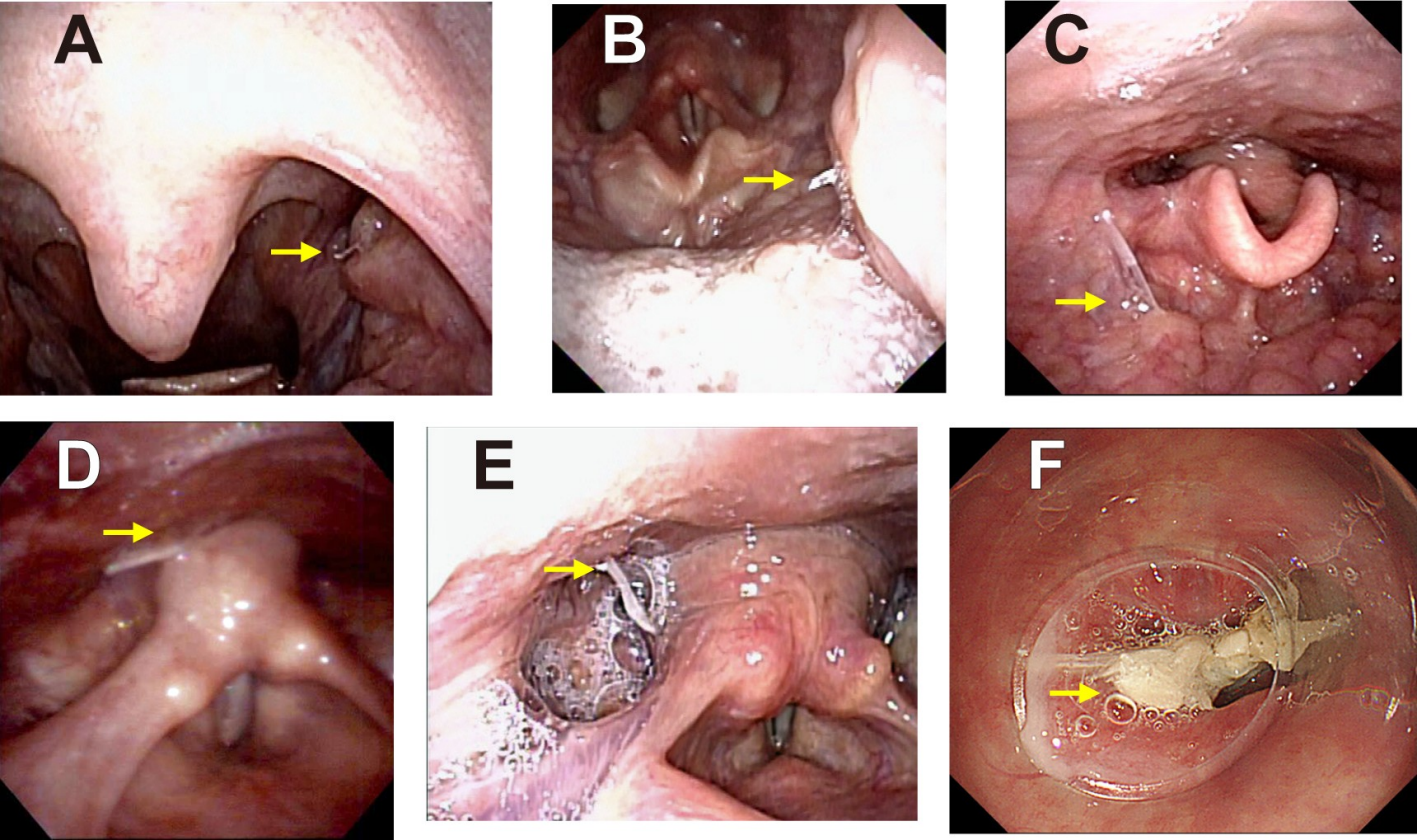
Typical images of fish bones in each location. A: herring bone in the palatine tonsil (upper pole), B: mackerel bone in the palatine tonsil (lower pole), C: flounder bone in the lingual tonsil (tongue base), D: amberjack bone in the posterior wall of hypopharynx, E: barracuda bone in the pyriform sinus, F: flounder bone in the cervical esophagus. Yellow arrows: fish bones.

In assessments based on the fish-bone foreign body location, 236 fish bones were found in the mesopharynx, which accounted for 87.4% of the cases (Table 1). In these 236 cases, the most frequently involved site was the palatine tonsil (n = 170), followed by the lingual tonsil (n = 49), the vallecula of the epiglottis (n = 9), the posterior wall (n = 5), and the pharyngoepiglottic fold (n = 3). Fifteen fish bones were discovered in the hypopharynx (5.6%), including the pyriform sinus (n = 8), the posterior wall (n = 6), and the postcricoid (n = 1). Nineteen fish bones were identified in the cervical esophagus (7.0%). Typical images of fish bones in each location are shown in Fig 3. Fish bone impaction was observed on the right side, left side, and the middle in 122, 115, and 14 cases, respectively, and the side of impaction was unknown in 19 cases.

**Table 1:** The locations of the fish-bone foreign bodies.

Mesopharynx (236)					Hypopharynx (15)			Esophagus (19)
Palatine tonsil	Lingual tonsil	Vallecula of the epiglottis	Posterior wall of the mesopharynx	Pharyngoepiglottic fold	Pyriform sinus	Posterior wall of hypopharynx	Postcricoid	Cervical esophagus
170	49	9	5	3	8	6	1	19

Table 2 shows the number of fish-bone foreign body cases. Fish species could not be confirmed in 16 cases (16/270, 5.9%). We also added ranking data for the consumption of fresh fish in each category (weight/household/year in 2017–2019) in Japan [17].

**Table 2:** The number of fish-bone foreign body cases and the ranking of household fresh fish consumption (weight/household/year in 2017–2019) in Japan (Statistics Bureau MoIAaC.

Name	Classification	Number [Total 270 cases]	Consumption ranking in Japan [17]
Japanese eel	Eel	34 (12.6%)	
Mackerel	Mackerel	33 (12.2%)	6
Salmon	Salmon	33 (12.2%)	1
Horse mackerel	Horse mackerel	30 (11.1%)	4
Righteye flounder	Flounder	28 (10.4%)	8
Saury	Saury	22 (8.1%)	5
Unknown	Unknown	16 (5.9%)	
Pacific cod	Pacific cod	10 (3.7%)	
Pacific Ocean perch	Pacific Ocean perch	10 (3.7%)	
Japanese amberjack	Amberjack	9 (3.3%)	3
Okhotsk atka mackerel	Others (large)	9 (3.3%)	
Red sea-bream	Others (large)	7 (2.6%)	10
Conger eel	Eel	3 (1.1%)	
Tuna	Others (large)	3 (1.1%)	2
Daggertooth pike conger	Eel	2 (0.7%)	
Lefteye flounder	Flounder	2 (0.7%)	
Sardine	Others (small)	2 (0.7%)	9
Herring	Others (small)	2 (0.7%)	
Croaker	Others (large)	2 (0.7%)	
Skipjack	Others (large)	2 (0.7%)	7
Japanese seabass	Others (large)	2 (0.7%)	
Great amberjack	Amberjack	1 (0.4%)	
Kichiji rockfish	Others (small)	1 (0.4%)	
Pond smelt	Others (small)	1 (0.4%)	
Spanish mackerel	Others (large)	1 (0.4%)	
Grunt	Others (large)	1 (0.4%)	
Barracuda	Others (large)	1 (0.4%)	
Red snapper	Others (large)	1 (0.4%)	
Carp	Others (large)	1 (0.4%)	
Blackthroat seaperch	Others (large)	1 (0.4%)	

Family Income and Expenditure Survey (2020)) [17].

We investigated the detection rate of fish bones in 368 cases with a suspected fish-bone foreign body (Table 3). The percentage of patients with no fish bones at the time of the visit and whose symptoms had resolved was 26.6%. However, among 33 cases with a suspected flounder-bone foreign body, fish bones were not found in only 3 cases (9.1%): thus, the flounder group contained a significantly lower proportion of cases in which the fish bone was not found at the time of the visit (3/33 for flounder bone cases vs. 90/335 for the remaining fish-bone cases; p = 0.0213). These results suggest that fish bones were not found at the time of the visit in 1/4 of the cases complaining of fish-bone foreign bodies, but flounder fish bones were significantly more frequent than others.

**Table 3:** Proportions of cases showing no fish bone or symptoms at the time of presentation.

Name	Total no. of cases	Cases showing no fish bone on presentation	Rate
Eel	47	8	17.0%
Mackerel	48	15	31.3%
Salmon	47	14	29.8%
Others (large)	39	8	20.5%
Flounder	33	3	9.1%
Horse mackerel	44	14	31.8%
Saury	31	9	29.0%
Unknown	23	7	30.4%
Amberjack	17	7	41.2%
Pacific cod	16	6	37.5%
Pacific Ocean perch	13	3	23.1%
Others (small)	10	4	40.0%
Total	368	98	26.6%

The number of cases without detectable fish bones was significantly lower in the flounder group (flounder vs the others, p = 0.0213).

**Fig 4:**
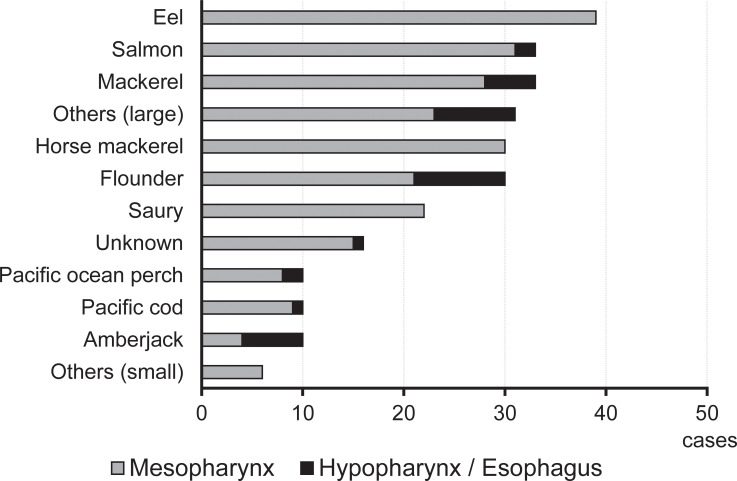
The locations (mesopharynx or hypopharynx/esophagus) of the fish bones for each fish type. The rate of hypopharynx/esophagus cases was significantly higher in the flounder group (flounder vs. the others, p = 0.0059).

We assessed the locations (mesopharynx or hypopharynx/esophagus) of the fish bones for each fish type. The mesopharynx was the most common location of fish bones in the study population, and all eel and saury bones were found in the mesopharynx (Fig 4). In contrast, flounder bones were frequently found in the hypopharynx/esophagus. In the comparison between flounder (mesopharynx:hypopharynx/esophagus, 21:9) and the others (215:25), the rate of hypopharynx/esophagus cases was significantly higher in the flounder group (p = 0.0059). These results suggest that while the bones of other fish generally stick in the mesopharynx, flounder bones tend to stick in the hypopharynx/esophagus.

**Fig 5:**
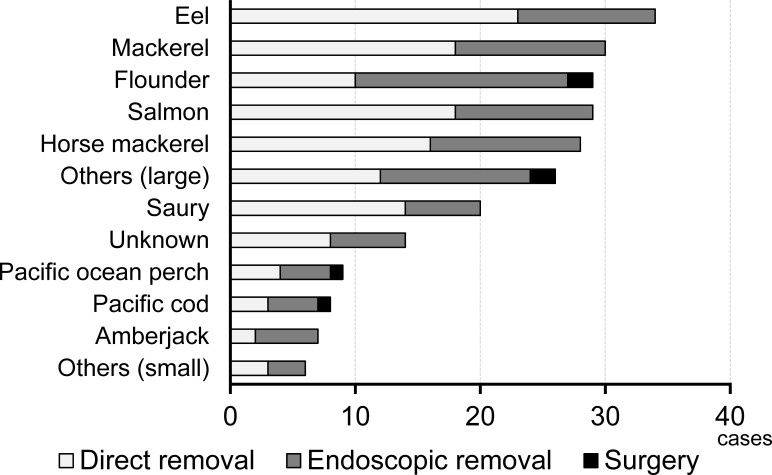
The removal methods of fish-bone foreign bodies. The proportion of cases involving direct removal was significantly lower in the flounder group (flounder vs. the others, p = 0.0278).

Next, we assessed the 240 cases in which fish-bone foreign bodies were actually removed by doctors. In 131 of these 240 cases (54.6%), the fish bone could be removed directly from the oral cavity with a tongue depressor and forceps, while the remaining 109 cases (45.4%) were treated by endoscopic removal (103 cases) or surgery (6 cases) (Fig 5). The conventional removal technique using indirect laryngoscopy with a mirror and forceps was not used in our hospital. Among the 29 cases involving flounder bones, only 10 (34.5%) could be treated with simple direct removal, and this rate was significantly lower than that for other fish bones (121 out of 211 cases, 57.3%) (p = 0.0278). Although most fish-bone foreign bodies were located in the mesopharynx, these results suggest that about 50% of fish-bone foreign body cases require special methods, including endoscopic removal or surgery.

Finally, we evaluated the differences between pediatric cases (<12 years old) and adult cases in relation to sex, location (mesopharynx or other locations), and removal methods (endoscopic removal/surgery or other methods) (Table 4). The 270 patients included 139 children aged under 12 years (72 males and 67 females; median age, 4 years; IQR, 3 to 8 years). The remaining 131 patients included 52 males and 79 females (median age, 51 years; IQR, 36 to 67 years). Fish bones were found in the mesopharynx in 138 out of 139 pediatric cases (99.3%) and 98 out of 131 older cases (74.8%). The detailed distribution and the rate of endoscopic or surgical removal in pediatric mesopharynx cases are as follows: palatine tonsil, 21 out of 123 cases (17.1%); lingual tonsil, 4 out of 8 cases (50%); vallecula of the epiglottis, 5 out of 5 cases (100%); posterior wall of the mesopharynx, 0 out of 1 case (0%); pharyngoepiglottic fold, 1 out of 1 case (100%). In total, 31 out of 139 pediatric cases (22.3%) required endoscopic or surgical removal, whereas 78 out of 131 older cases (59.5%) required endoscopic or surgical removal: the age-based groups showed significant differences in fish-bone location and the removal methods (p < 0.001 in both). These results suggest that most pediatric fish-bone foreign bodies are located in the mesopharynx and do not require special methods for removal.

**Table 4:** The differences between pediatric patients (<12 years of age) and older patients in relation to sex, location (mesopharynx or other locations), removal methods (endoscopic removal/surgery or other methods), and fish species.

	Pediatric patients (n = 139)	Older patients (n = 131)	p value
Median age (interquartile range, years)	4 [3–8]	51 [36–67]	
Sex (male:female)	72:67	52:79	p = 0.0512
Located in the mesopharynx	99.3% (138)	74.8% (98)	p<0.0001
Removal by endoscopic or surgical method	22.3% (31)	59.5% (78)	p<0.0001
Eel	18.7% (26)	9.9% (13)	
Salmon	14.4% (20)	9.9% (13)	
Mackerel	13.7% (19)	10.7% (14)	
Horse mackerel	13.7% (19)	8.4% (11)	
Others (large)	9.4% (13)	13.7% (18)	
Saury	9.4% (13)	6.9% (9)	
Flounder	6.5% (9)	16.0% (21)	
Unknown	5.8% (8)	6.1% (8)	
Pacific ocean perch	2.9% (4)	4.6% (6)	
Pacific cod	2.2% (3)	5.3% (7)	
Amberjack	2.2% (3)	5.3% (7)	
Others (small)	1.4% (2)	3.1% (4)	

## 4. Discussion

Accidental retention of fish bones in the upper aero-digestive tract is a common clinical problem in otorhinolaryngology practice in countries with high fish consumption, particularly in East Asia where a whole fish is often served without removing its bones [1, 2, 18]. In this study, we observed the following clinical characteristics in cases of fish-bone foreign bodies in the aero-digestive tract in Northeast Japan. First, the highest prevalence of these foreign bodies was in patients aged 0–4 years, and the majority of pediatric cases involved the mesopharynx, while the rate of pediatric cases requiring endoscopic or surgical removals was low (22.3%). Second, overall, 87.4% of the cases involved the mesopharynx, and the palatine tonsil was the most common site (63.0%). Third, fish-bone foreign bodies were usually caused by fish with high household consumption, except eel. Fourth, in 11.1% of the cases in which fish bones were identified on the first examination, the bones were dislodged during history-taking and subsequent examinations. Finally, flounder fish bones often lodge in the hypopharynx and esophagus, making it difficult for them to dislocate spontaneously and significantly increasing the need for endoscopic or surgical removals.

Understanding the sites at which bones of different fish species usually lodge is useful since these sites can be thoroughly assessed after history-taking. In this study, 236 of the 270 cases (87.4%) showed fish bones in the mesopharynx, especially in the palatine tonsil (63.0%). Several studies have reported that most fish bones were found in the mesopharynx as in our study [1, 11, 19, 20]. However, the percentages of different locations varied across reports, and Swain et al. reported that only 31.6% of their cases showed fish bones in the palatine tonsils [20]. These differences may be attributed to differences in age distribution since we have observed an apparent higher frequency of palatine tonsil impaction of fish bones in pediatric cases. Moreover, differences in the types of frequently consumed fish species in different regions and countries may contribute to differences in the sites of impaction. We found that eel, which is traditionally consumed in the summer season in Japanese culture, was the most common cause of fish-bone foreign bodies (39/270, 14.4%) in Japan, and its bones were mainly stuck in the mesopharynx. Although the overall household consumption of eel is not high, eel bones are tiny and numerous; therefore, it is difficult to remove its bones before eating. In Korea, croaker is the most common cause of fish-bone foreign bodies (63/286, 22.0%), whereas croaker fish-bone foreign bodies were only observed in 0.7% (2/270) of the cases in this study. Moreover, the difference in the methods used for preparing fish dishes in different regions and countries may also be related to the differences in the frequencies of various impaction sites, e.g., fish stew is most likely to cause esophageal foreign bodies [11]. However, our records did not include information regarding the fish recipes used in these cases, so we excluded this aspect from the investigation. Future studies should aim to evaluate the frequent fish-bone foreign body sites in relation to fish species and recipes from various regions and countries.

Fish bone can sometimes cause various severe complications, such as mesopharyngeal cellulitis, deep neck abscess, retropharyngeal abscess, thyroid abscess, mediastinitis, and perforation of the esophagus [13, 18, 21, 22]. Therefore, correct identification of the fish bone and its immediate removal is essential. We found that bones of eel, saury, horse mackerel, and other small fish, including sardine and herring, which are major fish species in Japanese food culture, were found in the mesopharynx in all cases. The bones of herring, sardine, and mackerel are minimally radio-opaque and difficult to identify on plain radiographs [23], so we can easily speculate that the bones of these small fish such as eel, saury, and horse mackerel are also radiolucent. In a case involving fish whose bones may easily impact the mesopharynx, negative radiography findings may not exclude the possibility of a foreign body. This indicates the importance of appropriate history-taking to identify the correct fish species, at least in Japanese patients, and suggests that similar studies in other cultures can provide valuable information specific to each culture. In addition, a CT scan, which is superior to plain radiographs in identifying fish bone [1, 24, 25], should be used to locate fish bones that are presumably radiolucent.

The method of removing the fish-bone foreign body varies depending on the location. An orally visible fish bone in the palatine tonsil can be easily removed directly. Fish bones in the lingual tonsil, the epiglottis’ vallecula, the hypopharynx, and the esophagus usually require flexible videoendoscopy: in some difficult cases, direct laryngoscopy or rigid esophagoscopy under general anesthesia may be required to remove the fish bones. The usefulness of a transnasal esophagoscope and a rigid curved laryngoscope in minimizing the scope of interventions has been reported in previous studies [24, 26]. In cases wherein the fish bone migrates to the extraluminal region in the upper digestive tract, a neck exploration surgery under general anesthesia through a transverse cervical incision may be required [18]. In our investigation, 109 patients (45.4%) required endoscopic or surgical removal, but none of the patients needed neck exploration surgery. Tang et al. have described neck exploration surgery for a migrating foreign body as “fishing for a needle in the deep ocean” [27], suggesting that this kind of surgery must be challenging. Thus, it is important to perform adequate preoperative assessment and effective imaging examinations during the operation, such as ultrasonography [18].

Among the foreign bodies caused by fish species, flounder-bone foreign bodies possess conspicuous characteristics, e.g., a tendency to lodge in the hypopharynx and esophagus, a tendency not to dislocate spontaneously, and the need for invasive techniques for foreign body removal. Flounder is a right or left-eyed flat fish with an oval body shape and variable coloration. It is distributed throughout the world in the northern hemisphere, including the Arctic Ocean, Pacific Ocean, Indian Ocean, and the Atlantic Ocean, and is widely consumed in various countries. In this study, the percentage of cases in which the fish bone was not found was 26.6% (98/368), and this result is consistent with the findings of a previous report from India (80/330, 24.2%) [20]. However, the corresponding rate in cases involving flounder bones (3/33, 9.1%) was significantly lower than those involving bones of other fish (95/335, 28.4%). Moreover, flounder fish bones required endoscopic or surgical removal more frequently. According to a report from Japan, flatfish (flounder) are the most common cause of esophageal fish-bone foreign bodies (5/11, 45.5%) [28]. Thus, if clinicians notice a flounder fish bone at the initial examination, they should recognize that it is unlikely to dislocate spontaneously and should proceed with more careful examination and treatment.

Pediatric patients frequently visit the emergency outpatient department or otolaryngology clinics for foreign bodies in the upper aero-digestive tract, such as fish bones, coins, and magnets [3, 29–31]. In our study, the numbers of children (<12 years old) and adults (≥12 years old) were almost the same, indicating no particular tendency for a greater proportion of pediatric cases involving fish-bone foreign bodies. We found that almost all the fish bones in pediatric cases were located in the mesopharynx, resulting in a lower proportion of endoscopic or surgical removals since we could remove fish bones in the mesopharynx directly from the oral cavity in many cases. Because the palatine tonsils are relatively large in children [3, 10], it is thought that the fish bone is likely to get caught there and does not move to the hypopharynx and the esophagus. However, because as much as 22.3% of the pediatric cases required endoscopic removal of fish bones, the general public should be thoroughly alerted about the risk of fish-bone foreign bodies and the importance of careful removal of fish bones while preparing fish foods for children.

## 5. Limitations

This study has some limitations. First, our study was a retrospective observational study using clinical records of fish-bone foreign body cases in a university hospital. Although our hospital is responsible for higher medical care, we also provide preliminary medical care for otolaryngological emergencies at night and on holidays. Thus, we believe our results reflect the current situation of fish-bone foreign bodies in Northeast Japan. Second, we obtained data for the household consumption of fresh fish from the database of Statistics Bureau, Ministry of Internal Affairs and Communications, Japan: this data does not accurately reflect the total fish consumption because it does not include fish consumption in restaurants or consumption of ready-cooked fish. Third, the trends in fish consumption and cooking methods differ depending on geographic regions and cultures: therefore, fish foreign bodies’ characteristics may also differ. However, many of the top fish species identified in this study, e.g., mackerel, salmon, flounder, and cod, are common worldwide, and we believe that the results of this study can be widely adapted to the characteristics of fish-bone foreign bodies in other parts of the world. Fourth, despite our thorough efforts, we could not obtain sufficient data on the specific co-morbidities of the patients, including the presence of dysphagia, size of fish bones, and cooking methods because of the retrospective study design. In addition, we could not examine fish with a small number of cases in detail. We found that 75.8% (182/240) of the information on fish bone size and 72.2% (195/270) of the information on cooking methods were missing in this study, so we did not perform further analyses. We acknowledge the possible presence of biases attributed to the lack of this information. Finally, outpatient follow-up was rarely performed in cases where fish bones were not found with or without CT scans and the symptoms disappeared. In cases where symptoms persist, a CT scan is recommended [2, 24]. However, in cases where symptoms disappeared, we considered that follow-up observations without further examinations, including CT scans, were sufficient, since foreign bodies can pass the intestinal tract uneventfully after entering the stomach [1], and most fish bones may get dissolved by stomach acids. Since perforation of the gastrointestinal tract can occur in sporadic cases, much attention should be paid to the appearance of abdominal symptoms after fish bone ingestion.

## 6. Conclusions

Fish bones in the upper aero-digestive tract are common foreign bodies encountered in the emergency department and otolaryngology clinics. In the present study, the characteristics of fish-bone foreign bodies were shown to differ depending on the fish species. Flounder bones often stick in the hypopharynx and the esophagus and are likely to require endoscopic or surgical removal. Moreover, in pediatric patients, almost all fish bones were found in the mesopharynx. Confirming the fish species in advance may be useful for appropriate diagnosis and treatment of cases with suspected fish-bone foreign bodies.

## Supporting information

S1 Data (XLSX)
